# Striving and thriving: Gender differences in the effects of climbing the socioeconomic ladder on stress and discrimination

**DOI:** 10.1016/j.socscimed.2025.118923

**Published:** 2025-12-31

**Authors:** Mitchell D. Wong, Benjamin P.L. Meza, Bridget Callaghan, Jennifer A. Silvers, A. Janet Tomiyama, Kimberly Narain, Rebecca N. Dudovitz

**Affiliations:** a Department of Medicine, David Geffen School of Medicine at UCLA, Los Angeles, Los Angeles, 90095, CA, United States; b Department of Psychology, University of California, Los Angeles, Los Angeles, 90095, CA, United States; c Department of Pediatrics, David Geffen School of Medicine at UCLA, Los Angeles, Los Angeles, 90095, CA, United States

## Abstract

**Background:**

Recent evidence suggests upward socioeconomic mobility (“striving”) may result in worse physical health. It has been hypothesized that striving might result in greater stress and discrimination, but prior observational evidence of this has been mixed.

**Methods:**

We analyzed data from The RISE UP study, a natural experimental study of 1270 low-income students in Los Angeles who were offered entrance to high-performing charter high schools via a random lottery. We compared lottery winners to those waitlisted, enabling objective assessment of striving with less confounding and selection bias. Self-reported stress using the Adolescent Stress Questionnaire and the Perceived Stress Scale and an abbreviated Everyday DiscriminationScale measuring lifetime discrimination were collected prospectively from adolescence into early adulthood.

**Results:**

During adolescence, perceived psychologic stress was greater among lottery winners than among the waitlist group but only among the females with little difference among males. During early adulthood, females exposed to high performing schools continued to report greater stress compared to waitlisted females. In contrast, among males, lottery winners reported lower stress compared to waitlisted subjects during this age period. No differences in perceived discrimination were observed between lottery winners and waitlisted students regardless of gender.

**Conclusions:**

These findings suggest that the process of upward mobility may incur psychological costs, particularly for females. The study highlights the need for further inquiry into mechanisms underlying gender disparities in the health effects of striving, and underscores the complex interplay between socioeconomic mobility, stress, discrimination, and health.

## 1. Introduction

Decades of research show lower socioeconomic status is linked to worse health (Bailey et al., 2017; Cutler and Lleras-Muney, 2006; T. J. Galama et al., 2018a; Lleras-Muney et al., 2024; M. Marmot and Bobak, 2000; M. G. Marmot et al., 1978; Williams et al., 2019), thus it seems intuitive that climbing the socioeconomic ladder (often referred to as “upward mobility” or “striving”) would improve health (“thriving”). Yet, recent evidence indicates that rising out of poverty may have negative physical health consequences for some. In a review of the literature, Chen and colleagues have described this phenomenon as “skin-deep resilience” referring to the observation that striving and upward mobility lead to outward improvements including better academic, behavioral, and socioeconomic outcomes but at the hidden expense of decrements in physiologic health (Chen et al., 2021). For example, an analysis of data from the Longitudinal Study of Adolescent to Adult Health (ADD Health) found African Americans adolescents from low-income families who were more engaged in school had better socioeconomic outcomes in adulthood, but they also had higher rates of diabetes compared to those who were less engaged in school (Brody et al., 2016).

Of note, the methods for operationally defining striving and upward mobility have varied across studies. These measures include self-reported measures of school engagement, self-control, and conscientiousness (Brody et al., 2016; Chen et al., 2019, 2020; Miller et al., 2016), between-generation improvements in education or income (Chen et al., 2024; Gaydosh et al., 2018; Miller et al., 2020), and teachers’ report of student self-control, academic ability, and social competence (Brody et al., 2013, 2020). Regardless of the measures used, several observational studies have supported this finding suggesting that striving may have negative cardiometabolic effects (Brody et al., 2013; Chen et al., 2020, 2021; Miller et al., 2020).

The paradoxical relationship between striving and thriving may only occur, or may be most pronounced, for certain groups. Prior studies show more school engagement, greater academic effort, and college attendance are linked to worse physical health among those who come from socioeconomically disadvantaged families (Brody et al., 2016; Chen et al., 2015). Furthermore, some studies have found that skin-deep resilience occurs only among minoritized groups, but not among striving whites (Brody et al., 2016; Chen et al., 2023; Gaydosh et al., 2018), while other studies have found improved socioeconomic status and factors associated with upward mobility, such as conscientiousness, are linked to worse physiologic health across racial groups including whites (Chen et al., 2020; Miller et al., 2020). Of note, most studies have focused on African Americans and whites, but some studies have observed skin-deep resilience among Latinx populations (Ehrlich et al., 2024; Gaydosh et al., 2018; Wong et al., 2022).

Few prior studies have examined sex differences in striving and thriving, but we have found that exposure to better schools leads to better health behaviors, worse self-reported physical and mental health only among females, but better physical health among males (Wong et al., 2022). Of note, studies of education interventions have shown that males benefit more than females regarding spillover effects on health (Campbell et al., 2014; Conti et al., 2016; T. Galama et al., 2018b; Heckman and Karapakula, 2019). For example, both the Perry Preschool Project and the Carolina Abecedarian Project tested the long-term impacts of preschool. The intervention improved educational and health outcomes for both males and females, but the health benefits were much greater among males than females (Conti et al., 2016). Thus, males may be protected against the negative effects of striving on health, but this finding is underexplored, and explanations are lacking.

The mechanisms underlying the paradoxical relationship between striving and thriving is not well understood, but some theorize that “weathering” may be a mediator (Chen et al., 2021; Geronimus, 1992). Specifically, climbing the socioeconomic ladder may increase chronic physiologic stress and allostatic load, leading to worse health outcomes later in life (Barton et al., 2024). Allostatic load refers to changes in the neurologic, neuroendocrine, and immune systems resulting from chronic stress (Fava et al., 2019; Lucente and Guidi, 2023; McEwen and Stellar, 1993), markers of which include elevated cortisol, epinephrine, norepinephrine, and blood pressure. These changes are linked to poverty and other forms of adversity, striving, and higher rates of chronic diseases including obesity, diabetes, cardiovascular disease, dementia, and cancer (Brody et al., 2013; Chen et al., 2015; Fuller-Rowell et al., 2012; Guidi et al., 2020; Ribeiro et al., 2018; Tian et al., 2020). The weathering explanation may also explain why some groups are more negatively affected by striving. For example, weathering may be worse for those who are disadvantaged because climbing the socioeconomic requires more effort and determination to achieve the same level of success as others in part due to structural and interpersonal racism and discrimination. This is referred to as “high-effort coping” (James, 1994; James et al., 1983).

Weathering may also involve greater psychological stress stemming from increased social isolation, social comparison, and a sense of deprivation relative to others that is exacerbated as individuals move between socioeconomic strata (Chen et al., 2021; Smith et al., 2012). This explanation could account for gender differences in striving and thriving. Females are more likely than males to experience expectations around caregiving that result in conflicting role obligations (Heise et al., 2019), and perhaps this conflict is magnified in the context of striving. Furthermore, high-effort coping is thought to emerge from a place of disadvantage. Thus, the intersection of having fewer economic resources, being minoritized, and female may lead to higher risk for discrimination, exposure to adversity, and competing or unmet needs (Chinn et al., 2021; Lin and Liu, 2023; Mersky et al., 2021). Females are also more attuned to social interactions than men, thus moving up the socioeconomic ladder may lead to more disruption of peer networks and social support (Bedrov and Gable, 2023; McMillan et al., 2018).

Evidence that striving leads to more psychological stress is mixed. The relationship of striving and perceived stress has been examined in only a couple of cohort studies and no link was found (Miller et al., 2020). Other studies have examined “goal-striving stress” and found a relationship to higher rates of chronic diseases including hypertension, heart disease, kidney disease, and mental health problems (Cain et al., 2019; Cain-Shields et al., 2022; Glover et al., 2019; Neighbors et al., 2011). However, goal-striving stress in these studies was measured by failure to achieve one's goals and the amount of disappointment that ensued (Sellers and Neighbors, 1999). Thus, these studies examined stress due to failure rather than stress related to striving. Furthermore, numerous studies have consistently shown that striving is associated with fewer rather than more depressive symptoms (Brody et al., 2013, 2016; Chen et al., 2020, 2024; Gaydosh et al., 2018; Miller et al., 2020). While several studies have found minoritized persons experience more perceived discrimination (Bather et al., 2024), we are not aware of studies that have examined the relationship between striving and discrimination.

The prior literature on striving and thriving have had two major limitations. First, prior research has been based almost entirely based on observational studies (Chen et al., 2021), thus raising questions about confounding and selection bias. Second, as mentioned above, the operational definition of striving and upward mobility varies greatly. Many measures are self-reported (e.g. school engagement), and/or subjective (e.g. teacher reported academic abilities and social competence). Many of these measures are likely to be confounded with other factors that influence health. For example, it is not surprising that students with more self-control, conscientiousness, and school engagement have better academic and socioeconomic outcomes and lower rates of depression and substance use. But it is also probably true that early childhood protective factors such as greater social support, stable family structure, and more effective parenting are potential confounders that lead to both striving and thriving. It is not clear that confounding would explain why socioeconomic mobility is linked to worse physiologic health; however, without more rigorous study designs and methods, it is difficult to determine the causal nature between striving and upward mobility with downstream consequences and explore potential mechanisms.

To better understand the causal impact of striving on thriving, we conducted the Reducing Inequities through Social and Educational Change (RISE Up) Study. This is an on-going natural experiment comparing a cohort of mostly Latinx adolescents from low-income neighborhoods of Los Angeles who applied to high-performing, public charter high schools in 2013 and 2014 (Dudovitz et al., 2018; Wong et al., 2022). We studied high-performing charter high schools that were over-subscribed (more applicants than seats available) and admitted students based on a random lottery. This allowed us to recruit applicants to these schools for participation in our study and observe comparable groups of low-income students exposed to higher- and lower-performing schools with less potential for confounding bias. In addition, our definition of striving is objective and extrinsically determined by the outcome of the lottery thus reducing selection bias.

As hypothesized, we found lottery winners had better standardized test results, took more rigorous coursework, and were more likely to attend college than lottery waitlisters (Reber et al., 2024). We also found that lottery winners had higher rates of obesity and overweight and worse self-reported physical and mental health, but only among females (Wong et al., 2022). Among males, exposure to higher-performing schools was linked to better physical health outcomes and no differences in mental health.

In the present study, we extend our analysis of the RISE Up natural experiment to examine the relationship between exposure to high-performing schools with perceived psychological stress and discrimination using data collected longitudinally from age 14 through age 22. Furthermore, we assess for a differential relationship among these variables across gender. We hypothesize that boys may have less psychological stress than girls and less exposure to discrimination in the context of high-performing school environments.

## 2. Methods

### 2.1. Sample and study design

As previously reported (Dudovitz et al., 2018; Wong et al., 2022), in the spring of 2013, we identified all public charter schools in Los Angeles that scored in the top tertile of academic performance measured by the 2012 Academic Performance Index (API), which is based on standardized test scores, attendance, and graduation rates. From this list, we identified those charter schools that were in low-income neighborhoods and had at least 50 more applicants than available seats. As required by California state law, public charter schools must admit students by random lottery if there are more applicants than seats available. Five charter schools met these criteria and all five agreed to participate in the study. For each school, we obtained a list of applicants and recruited participants from the list of lottery winners and waitlisted students. We attempted to contact 1995 charter school applicants at the transition to grade 9 to participate in the study. Three hundred nineteen could not be contacted, and of the remaining 1676 whom we contacted, 239 refused participation and 167 were ineligible for the study because they had moved out of the area or were admitted outside the lottery because their sibling was already attending the school. The final sample included 1270 students (694 lottery winners and 576 lottery waitlist). We obtained assent for participation from each student and informed written consent from each student's parent/guardian.

All 1270 students completed a baseline survey at the transition to grade 9. Annual retention rate in the study exceeded 95 % in all years of the study and was similar between the lottery winners and waitlist groups. All five intervention schools served low-income neighborhoods with predominantly Latinx families and adopted a similar school model that included smaller class size, high academic standards for all students, and an expectation of college matriculation (Rohn, 2024).

Data for this study is publicly available at https://tinyurl.com/53r75r97. All analyses were conducted using Stata SE 19.5.

### 2.2. Measures

At each survey, except for a brief survey conducted in grade 12, we assessed self-reported stress. For surveys conducted in grade 9 (baseline), grade 10, grade 11, and at age 20 (approximately 2 years after high school graduation), students completed the Adolescent Stress Questionnaire (ASQ), which contained 27 items (Byrne et al., 2007). The ASQ stress score includes the total (combined) stress measure as well as components including stress related to home (6 items), school performance (4), romantic partner (4), peers (4), future (3), school-leisure balance (3), and money (3).Cronbach's alpha for the total ASQ stress scores ranged from 0.92 to 0.93 and from 0.80 to 0.91 for the ASQ stress subscales. At age 21 and 22 surveys, we assessed stress using the 10-item Perceived Stress Scale (PSS), which is validated for use in adults (Baik et al., 2019; Cohen et al., 1983). Cronbach's alpha was 0.78 and 0.84, respectively.

At the age 21 survey, we measured lifetime perceived discrimination using the short version of the Everyday Discrimination Scale, which asked respondents to report if they had ever experienced the following: others treated you with less courtesy, provided worse service to you, treated you as stupid, acted afraid of you, treated you as dishonest, and acted superior (Williams et al., 1997). We counted the number of items to which respondents responded “yes” (alpha 0.81). Subjects were also asked if the experience was due to ancestry, gender, race, age, height or weight, or some other physical appearance.

Sociodemographic measures were gathered in the baseline survey and were all self-reported by the adolescent. These included gender (male, female, other), race/ethnicity, country of birth, native language, and parental demographics (country of birth, employment, and education attainment). We use the term gender rather than sex because that is how we asked the question in our survey. Given the distribution by race/ethnicity in the sample, respondents were categorized as Latinx versus non-Latinx.

Middle school grade point average (GPA) was obtained from academic transcripts when available (n = 1044, 82 % of the sample) or from self-report at baseline survey if transcripts could not be obtained (n = 198, 16 %).

### 2.3. Analysis

We conducted intent-to-treat analyses comparing lottery winners and waitlist students. We used three-level hierarchical regression models that adjusted for repeated measures of the outcome (stress) over time within individual respondents and multiple respondents clustered within the same school. The mixed models examining the discrimination outcome just had two-levels since discrimination was only measured at one time point. Time was coded as a categorical variable (e.g. grade 9, grade 10, etc.). All models included random intercepts at the student and school levels and random slopes of time to vary for each student, capturing individual growth curves or trajectories over time within students. The mixed command in STATA version 17 (College Station, TX) was used for all regression analyses.

Each model included 3-way interaction terms between the main exposure variable (lottery winner vs. waitlist), student gender (male vs. female) and time (wave of survey). Although students were given a third option to report their gender as “other”, all students reported their gender as either male or female. We also adjusted for students’ Latinx ethnicity, preferred language (English vs. other), and birthplace (U.S. vs foreign-born). Latinx ethnicity was included in the model as a marker of shared culture and experiences rather than a biological characteristic. Given that Latinx ethnicity, native language, and birthplace may also be proxies for experiencing discrimination, we performed a model without controlling for these variables. However, this sensitivity analysis had almost no effect on the analyses, so for simplicity, all reported models included these covariates. We also adjusted for GPA in grade 8, parental birthplace (at least one born in U.S. vs. both foreign-born), parental education (at least one graduated from high school vs. other), and parental employment (at least one parent full-time employed vs. other). Twenty-eight students with missing transcripts and no self-reported scores were categorized as “missing GPA” and kept in the model. Finally, we adjusted for the combination of charter schools that students applied to, referred to as the risk set. This method of adjustment has been used in prior charter school natural experiments and accounts for the possibility that students who apply to more than one charter school would have a higher chance of being a lottery winner (Abdulkadiroğlu et al., 2011; Angrist et al., 2013).

We report estimated differences in outcomes (stress and discrimination) between lottery winners and waitlist for males and females. Because we used different measures of stress before age 21 (ASQ) and after age 21 (PSS), we conducted separate regression models for perceived stress during adolescence and during early adulthood. To enhance interpretability and comparison of the two models, we standardized ASQ and PSS scores so that a 1-point change is equal to 1 standard deviation. Estimated standard errors were computed using bootstrapping methods with clustered sampling at the participant-level (1000 replicates). P values for the interaction terms are reported directly from the hierarchical regression models.

## 3. Results

### 3.1. Baseline characteristics of lottery winners and waitlisted participants

By age 22, 514 (74.1 %) lottery winners and 401 (69.6 %) of those on the waitlist completed the 6th follow up survey (*p* = 0.08). Table 1 shows student and parental characteristics stratified by gender and admission lottery results. The lottery winners and waitlisted participants were similar in most baseline characteristics. Among females, lottery winners were more likely than waitlisted participants to be Latina (91.9 % vs 86.3 %, *p* = 0.02) and were less likely to have middle school transcripts (78.6 % vs. 89.3 %, *p* < 0.001).

### 3.2. Exposure to high-performing schools and self-reported stress

Table 2 shows the sample size and median age at each survey wave. It also shows the range and mean stress scores standardized so the standard deviation equals 1.

Adjusted for baseline demographics, parental characteristics and GPA in middle school, overall perceived stress was greater among lottery winners than among the waitlist group but only among the females (Table 3). Between ages 14–20, lottery winners reported higher standardized Adolescent Stress Questionnaire (ASQ) scores than waitlisted participants – a difference of 0.148 in total stress (95 %CI: 0.046 to 0.249) among females, but only 0.045 (95 %CI: −0.052 to 0.143) among males. At age 21 and 22, the standardized Perceived Stress Scale (PSS) score was higher among lottery winners than waitlisted females (winner vs. waitlist difference = 0.137, 95 %CI: 0.013–0.261) but was lower among lottery winners than waitlisted males (difference = −0.130, 95 % CI: −0.274 to 0.014). The test for an interaction effect between exposure to high-performing schools and gender was statistically significant for the stress model at age 21 and 22 (*p* = 0.02), but not for stress model at age 14–20 (*p* = 0.22). These gender differences in stress response between lottery winners and waitlisted was observed for stress at home, stress of peer pressure, and stress of financial pressure. For stress related to schoolwork (stress about school performance and stress about conflicts between school and leisure), lottery winners reported more stress than waitlisted students, and these findings were similar for males and females.

**Fig. 1.**
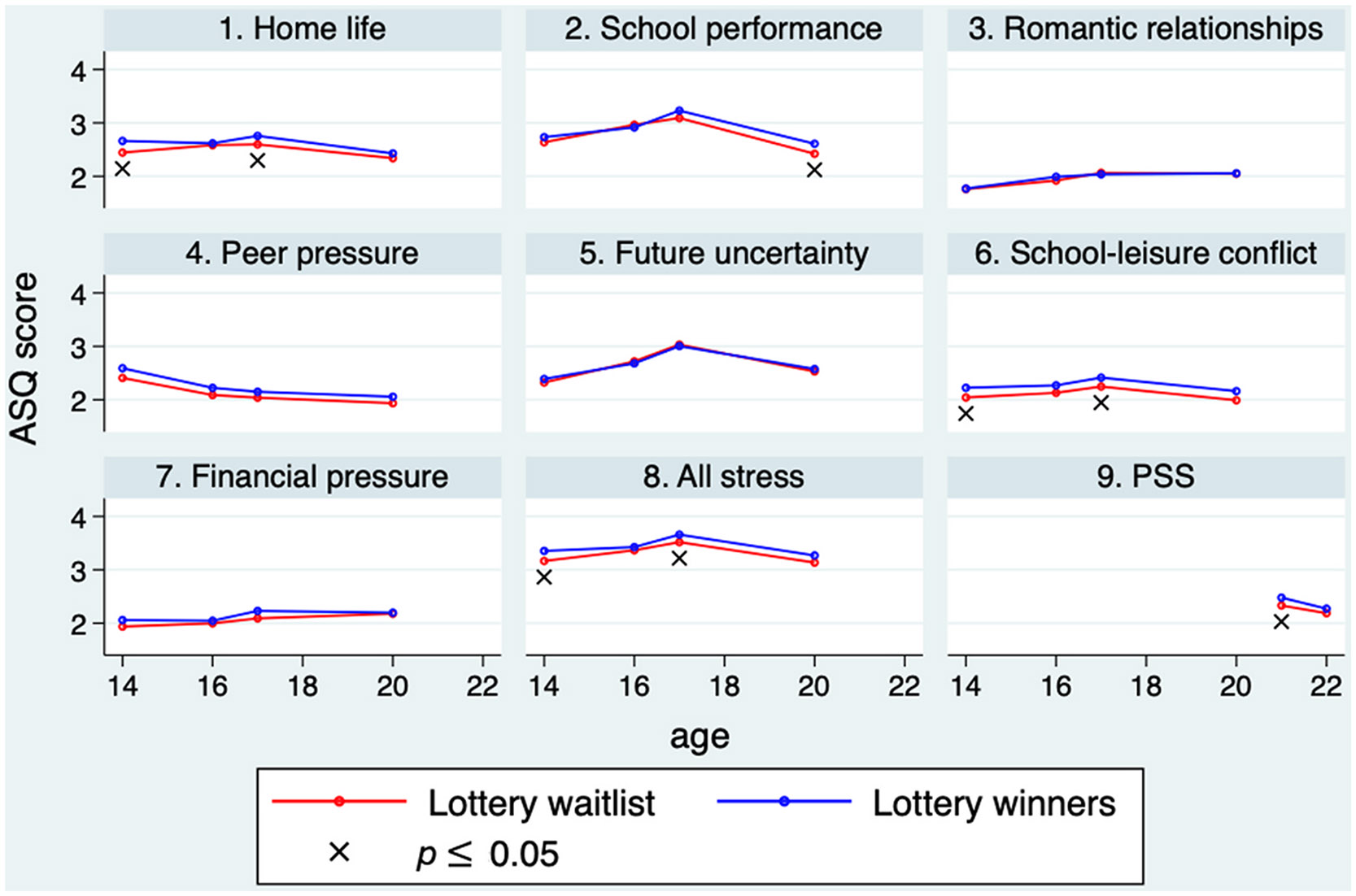
Stress scores among females.

**Fig. 2.**
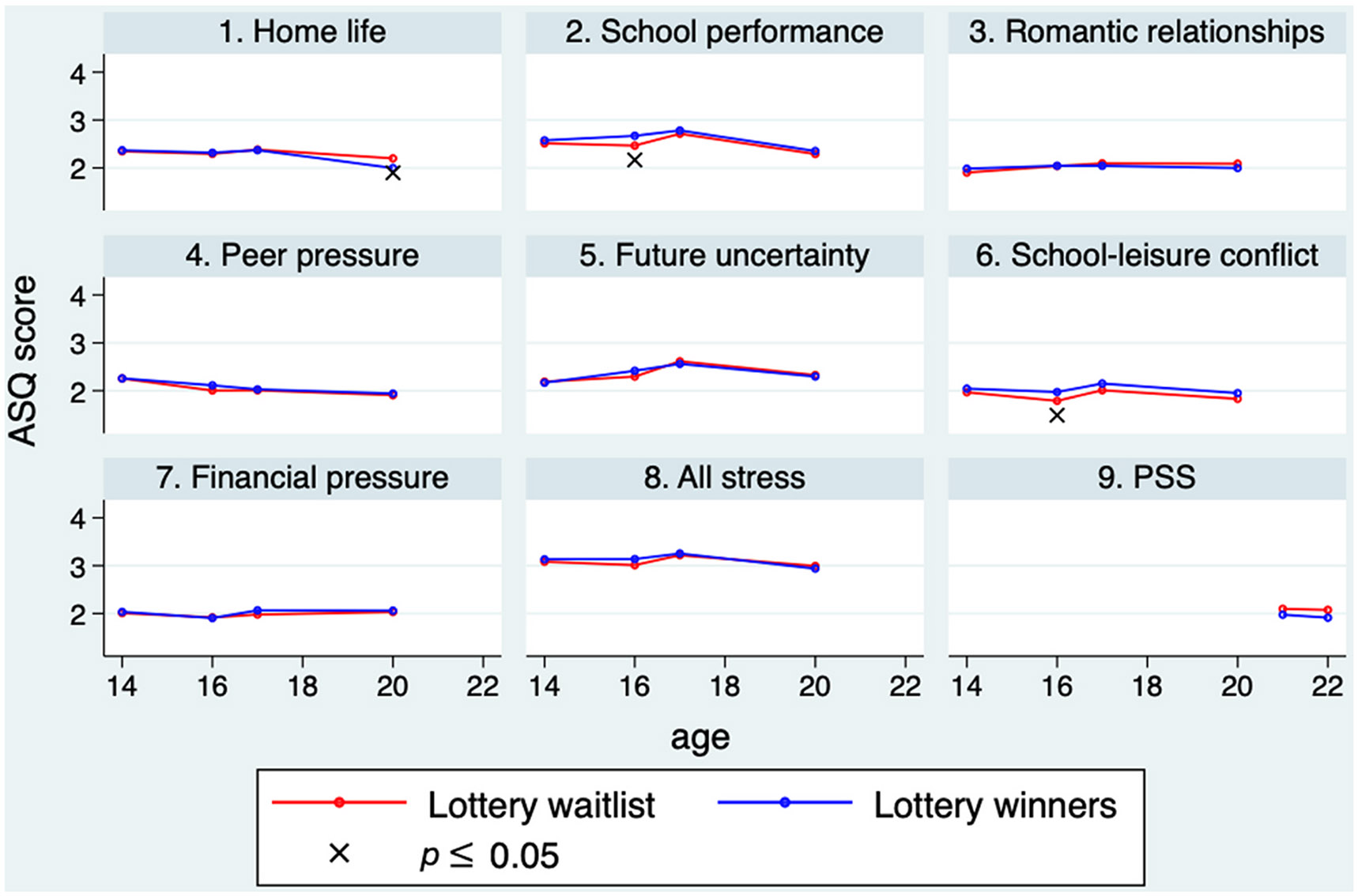
Stress scores among males.

Graphs of stress scores over time are shown for lottery winners and waitlisted females (Fig. 1) and males (Fig. 2). At any specific time point, the difference between winners and waitlisted groups was modest. Overall stress, stress about school performance, and stress about the future peaks for all participants at age 17 (grade 11). Note that we did not assess stress in grade 12. Stress about peer pressure was highest at baseline (grade 8-grade 9) and then declined (*p* < 0.001 for each comparison at grade 10, 11 and age 20 compared to baseline).

### 3.3. Exposure to high-performing schools and perceived discrimination

We compared perceived discrimination between lottery winners and waitlisted participants by gender (Table 4). Using the Everyday Discrimination Scale, participants reported if they had ever been treated differently due to gender, race, age, height or weight, other physical appearances, other reasons, or no discrimination. The unadjusted proportion reporting discrimination for any reasons, due to gender, and due to race is shown for each of the 6 items comprising this scale. Comparing lottery winners and waitlisted, we found no significant differences among females or males for any of the items. Of note, females were much more likely to report discrimination compared to males for any reason, due to gender, and due to race. For example, 49 % of females (lottery winners and waitlisted combined) reported being treated with less courtesy for any reason compared to 31 % of males (*p* < 0.001). The difference was even larger when differential treatment was attributed to gender (12.5 % among females vs. 0.9 % among males, *p* < 0.001) and smaller when treatment was due to race (20.3 % among females vs. 15.6 % among males, *p* = 0.06). Similar patterns were observed for receiving poorer service, being treated as stupid, and others acting superior.

We also examined mean scores on the Everyday Discrimination Scale, calculated as summation for each item (1-point each). We examined the mean scale scores being treated differently for any reason, due to gender, and due to race (Table 5). We found no significant differences in these mean scores by lottery assignment among males or females, in unadjusted analyses or analyses adjusted for baseline student and parental characteristics. However, in both unadjusted and adjusted analyses, females reported higher discrimination scores for any reason, due to gender, and due to race (*p* < 0.001 for all comparisons).

Since Latino ethnicity, language, and birthplace may be closely correlated with experiences with discrimination or sources of stress, we conducted sensitivity analyses excluding these covariates in our models. The results from the sensitivity analyses (Appendix Tables 6 and 7) were similar to those from the models that included these covariates.

## 4. Discussion

In the present study, we used a natural experiment to examine exposure to high-performing schools and its relationship with stress and perceived discrimination. We found lottery winners, compared to those waitlisted, had greater self-reported stress but only among females. We found no differences between lottery winners and waitlisted in perceived discrimination. The observed gender differences in stress in this study follow a similar pattern in the Jackson Heart Study, which found women reporting greater goal-striving stress than men (Cain-Shields et al., 2022; Glover et al., 2019). As previously noted, goal striving stress is defined as the disappointment associated with not achieving one's goals. In the RISE Up Study, we defined striving based on admission by random lottery to high-performing schools and thus less likely confounded by factors that influence one's aspirations or likelihood of success. We found overall differences in stress between lottery winners and waitlisters were statistically significant but quite small. However, this is consistent with the weathering hypothesis that small amounts of greater stress over a long period of time might result in widening differences in health outcomes with age (Geronimus, 1992).

We were unable to determine why lottery winners reported more psychological stress, but we should first consider the general psychological stresses that may have been created by attending a high-performing school. The RISE Up study recruited students applying to “no excuse” charter schools, which was the most adopted model among early charter schools and emphasized strict disciplinary rules and adherence to high academic expectations and for all students (Krowka et al., 2017). Academic rigor relied on standardized tests as an important measure of performance and a focus on college preparation. From qualitative interviews of RISE Up participants, we found attending high-performing charter schools shifted student's academic and behavioral self-concept, which may have led to greater psychological stress (Dudovitz et al., 2017). We found that lottery winners, compared to waitlisted students, reported greater stress about academic performance and conflicts about school/leisure. This finding was observed for both males and females and likely reflects the high expectations that these charter schools set for all students. In addition, charter school students were required to wear uniforms, which was intended to foster a new school culture separate from a traditional public school. However, this physical and cultural separation of charter schools from local neighborhood schools may have created internal psychological conflict as well as conflict with peers in the neighborhood who did not attend charter schools. For example, charter school students may have been accused of “acting white,” a potential consequence for students from lower socioeconomic and minority backgrounds who prioritize behaviors that are perceived to lead to success within the culture in power, such as academic performance and college attendance, over fitting in with one's peers (Fordham and Ogbu, 1986).

Why females and males have different psychological stress is unclear. One possible explanation for this difference could be related to the observation that females tend to perceive more risk than males and are more risk averse (Boholm, 1998; Larkin and Pines, 2003). They also report more anxiety than males when faced with uncertainty (Panchyshyn et al., 2023). Sex differences in risk taking may be due to the influence of testosterone (Nofsinger et al., 2018), which is linked to increase impulsivity and sensation seeking (Kuhn, 2015). Thus, females might experience high-performing schools with a greater sense of uncertainty about the future, perceive uncertainty with greater distress, and be more biologically sensitive to stress compared to males, particularly during adolescence (Hodes and Epperson, 2019). Of note, we previously examined general anxiety in our RISE Up cohort and found female lottery winners had slightly higher prevalence of moderate to severe anxiety compared to waitlisted females, and male lottery winners had lower prevalence of anxiety compared to waitlisted males. However, these differences were not statistically significant (Wong et al., 2022).

When examining types of stress, we found gender differences occurred with stress at home. Females may have different roles and expectations than males. Among Latinx families, this difference has been referred to as “Marianismo”– a term derived from the idealization of the values of the Virgin Mary including self-sacrifice and family (Pinã-Watson et al., 2016). Thus, perhaps females in our study had more traditional expectations that they take on duties at home related to chores and caregiving. Female lottery winners in our study also reported more stress related to peer pressure than waitlisted females, which is consistent with prior studies that have found females are more sensitive to peer pressure than males (Geven et al., 2017).

Although females in our study were generally more likely to report discrimination than males, we did not observe that lottery winners reported more discrimination than waitlisted students among males or females. Of note, we did not assess the degree to which experiences of discrimination were distressing. Therefore, we do not know whether discrimination stress varies by gender. It is also possible that the frequency of discrimination or types of discriminatory experiences lead to increased stress and/or worse health outcomes and that these effects vary by gender. Future studies should examine these more nuanced aspects of the potential relationships among striving, discrimination, gender and thriving.

The study has advantages compared to other studies on this topic. Foremost is our measurement of striving, which is broadly defined as climbing the socioeconomic ladder (Chen et al., 2021) but operationally defined in varied ways. Some prior studies have studied the personality traits and individual characteristics that increase the likelihood of upward mobility (e.g. conscientiousness, engagement in school, academic competence, and educational aspirations). These measures, however, have inherent limitations including subjectivity, measurement bias, and confounding from the influence of parents, peers, and upbringing. Other studies have examined the successful outcome of striving (e.g. college graduation and improved socioeconomic status in adulthood compared to childhood), but this measure is also likely confounded by parents and unobserved opportunities and influences. In contrast, we examined exposure to higher performing high schools, i.e. the opportunity to climb the socioeconomic ladder. This definition not only avoids subjective and measurement bias, but it also permits us to perform a natural experiment, which minimizes confounding and selection bias. Because of this study design, we can more confidently make inferences about causality.

### 4.1. Limitations

Despite the strengths of our study, a few limitations are worth noting. Our sample primarily consisted of Latinx adolescents in Los Angeles; thus, we are unable to determine if our findings are generalizable to other racial and ethnic groups or teens in other geographic areas. Prior studies have noted an inverse relationship between striving and thriving among African Americans, but not among whites (Chen et al., 2021). While our study is the first to our knowledge to examine this question among a predominantly Latinx population, we do not have a sample size large enough to determine whether the relationship between striving and stress differed by ethnicity. We also measured self-reported stress and do not have any biological measures of stress response. We were unable to use the same measure of stress in adolescence and adulthood. The Adolescent Stress Questionnaire is specifically designed for adolescents with stressors anchored to that developmental stage and do not apply in adulthood (e.g., "Following rules at home," and "Not having enough time for activities outside of school") (Byrne et al., 2007). However, we chose a very well-validated and widely used measure of perceived stress in adulthood using the Perceived Stress Scale (Baik et al., 2019; Cohen et al., 1983), and both measures capture subjective self-reported stress appraisals. Lastly, the present study analyzed data collected through early adulthood. Accumulated differences in stress may continue to widen over time and with age through middle and later adulthood.

## 5. Conclusion

A growing body of evidence not only indicates that climbing the socioeconomic ladder has become more difficult (Chetty, 2021; Chetty et al., 2014a; Chetty et al., 2014b), but doing so may also have negative effects on health despite the fact that higher socioeconomic status is clearly linked to fewer chronic diseases and lower mortality (Cutler and Lleras-Muney, 2006; T. Galama et al., 2018b; Lleras-Muney, 2022; Wong et al., 2002). We have yet to understand why striving may lead to worse health, but our study is consistent with the weathering hypothesis and the potential role of greater stress as a consequence of upward mobility, particularly among females. It remains unclear why striving may have negative effects on thriving and why there are gender differences in this relationship. Possible explanations include unrecognized negative behavioral, societal, or biological processes that result from striving, which may also be connected to gender or sex differences in the effort to strive, the associated experience of stress or stressors, and/or the context of structural disadvantage. Understanding why females are more vulnerable to the negative effects of striving (or why males are more protected) may be key to understanding how to improve upward mobility and health simultaneously for all persons.

## Supplementary Material

1

## Figures and Tables

**Table 1:** Comparison of lottery waitlist and winners, n (%).

	Females			Males		
	Lottery waitlist	Lottery winners	P value	Lottery waitlist	Lottery winners	P value
**N**	299 (44.8%)	369 (55.2%)		277 (46.0%)	325 (54.0%)	
**Latino ethnicity**	258 (86.3%)	339 (91.9%)	0.020	242 (87.4%)	295 (90.8%)	0.180
**US born**	262 (87.6%)	322 (87.3%)	0.888	243 (87.7%)	286 (88.0%)	0.918
**English native speaker**	113 (37.8%)	140 (37.9%)	0.969	120 (43.3%)	146 (44.9%)	0.693
**At least 1 parent born in US**	79 (26.4%)	103 (27.9%)	0.667	75 (27.1%)	79 (24.3%)	0.438
**At least 1 parent graduated high school**	149 (49.8%)	195 (52.8%)	0.439	142 (51.3%)	180 (55.4%)	0.312
**At least 1 parent working full time**	251 (83.9%)	324 (87.8%)	0.152	242 (87.4%)	288 (88.6%)	0.637
**Middle school GPA**						
<2.0	21 (7.0%)	29 (7.9%)	0.690	57 (20.6%)	46 (14.2%)	0.126
2.0–2.5	57 (19.1%)	58 (15.7%)		62 (22.4%)	70 (21.5%)	
2.6–3.0	68 (22.7%)	75 (20.3%)		73 (26.4%)	85 (26.2%)	
3.1–3.5	77 (25.8%)	97 (26.3%)		44 (15.9%)	71 (21.8%)	
3.6–4.0	69 (23.1%)	102 (27.6%)		33 (11.9%)	48 (14.8%)	
Missing transcripts and self-reported GPA	7 (2.3%)	8 (2.2%)		8 (2.9%)	5 (1.5%)	
**Middle school transcripts obtained**	267 (89.3%)	290 (78.6%)	<0.001	232 (83.8%)	255 (78.5%)	0.10

**Table 2:** Study sample age and the standardized mean and range of the stress measures.

Survey	Baseline	1	2	4	5	6
**Grade**	9	10	11	1 year after high school	2 years after high school	3 years after high school
**Median age**	14.2	15.8	16.7	19.7	20.8	22.3
**N**	1270	1158	1114	997	961	914
**Stress measure**	ASQ	ASQ	ASQ	ASQ	PSS	PSS
Standardized mean	3.19	3.24	3.42	3.1	2.24	2.12
Range	1.61–7.02	1.61–6.72	1.61–7.73	1.61–8.03	0–5.26	0–5.71

ASQ = Adolescent Stress Questionnaire; PSS=Perceived Stress Scale.

Mean stress scores are standardized to a standard deviation equal to 1. Survey follow-up 3 was a brief survey conducted at grade 12 and did not assess stress.

**Table 3:** Differences (se) in stress between the lottery winners and waitlist groups among females and males.

	Females	Males	All	P value for gender*lottery winner/ waitlist interaction term
**ASQ Total Stress at age 14–20**	0.148** (0.052)	0.045 (0.05)	0.1** (0.037)	0.22
Stress at home	0.154** (0.052)	−0.018 (0.046)	0.073* (0.036)	0.08
Stress about school performance	0.091 (0.046)	0.086 (0.048)	0.089** (0.034)	0.75
Stress about romantic relationships	0.012 (0.044)	0.02 (0.046)	0.016 (0.033)	0.50
Stress of peer pressure	0.149** (0.054)	0.021 (0.047)	0.089* (0.037)	0.14
Stress of future uncertainty	0.028 (0.045)	−0.002 (0.048)	0.014 (0.034)	0.43
Stress about the school/leisure conflict	0.169** (0.048)	0.114* (0.049)	0.144** (0.035)	0.35
Stress of financial pressure	0.096* (0.049)	0.03 (0.049)	0.065 (0.036)	0.39
**Perceived stress scale at age 21 and age 22**	0.137* (0.063)	−0.13 (0.073)	0.012 (0.049)	0.02

Intent-to-treat analyses were used to estimate difference in stress between lottery winners vs. waitlist. Hierarchical regression models account for multiple observations over time and students clustered within schools and include interaction terms between the main exposure (lottery winners vs. waitlist) and gender. Standard error estimates were based on bootstrapping with 1000 repetitions. Significance levels

* = 5 %

** = 1 %.

**Table 4:** Unadjusted proportion reporting "yes" for each item of the Everyday Discrimination Scale.

Discrimination	Females				Males			
	Lottery winners	Waitlist	Difference	*p value*	Lottery winners	Waitlist	Difference	*p value*
**Treated with less courtesy**								
For any reason (%)	52.2	44.8	7.4	0.09	33.3	27.3	6.1	0.18
Due to gender (%)	12.2	12.9	−0.7	0.80	0.8	1.1	−0.3	0.79
Due to race (%)	21.7	18.5	3.2	0.37	16.9	13.9	3.0	0.40
**Received poorer service**								
For any reason (%)	41.2	37.5	3.7	0.39	21.4	19.8	1.6	0.68
Due to gender (%)	3.0	3.5	−0.4	0.79	0.8	0.0	0.8	0.21
Due to race (%)	23.0	22.8	0.1	0.97	14.4	7.5	6.9	0.03
**Other treated you as stupid**								
For any reason (%)	35.3	41.0	−5.7	0.18	20.6	19.8	0.8	0.84
Due to gender (%)	11.9	18.5	−6.7	0.03	0.0	1.6	−1.6	0.05
Due to race (%)	8.8	6.9	1.9	0.42	6.2	7.0	−0.8	0.75
**Others acted afraid of you**								
For any reason (%)	20.3	16.8	3.5	0.30	19.0	22.3	−3.3	0.40
Due to gender (%)	2.4	3.0	−0.7	0.65	1.2	3.2	−2.0	0.16
Due to race (%)	9.8	9.1	0.8	0.76	6.2	5.3	0.9	0.70
**Treat you as dishonest**								
For any reason (%)	19.0	19.0	0.0	1.00	11.6	15.5	−3.9	0.23
Due to gender (%)	3.1	6.0	−3.0	0.10	1.2	2.1	−0.9	0.47
Due to race (%)	7.8	6.5	1.3	0.56	5.4	6.4	−1.1	0.65
**Others acted superior**								
For any reason (%)	56.8	51.7	5.0	0.25	26.5	29.4	−3.0	0.50
Due to gender (%)	17.6	13.8	3.8	0.24	0.8	1.1	−0.2	0.80
Due to race (%)	17.6	21.6	−4.0	0.25	11.6	12.8	−1.3	0.69

**Table 5:** Everyday Discrimination Scores for the lottery winners and waitlist groups stratified by gender.

	Females				Males			
		
	Lottery winner	Lottery Waitlist	Difference	*p* value for gender* lottery winner/ waitlist interaction term	Lottery winner	Lottery Waitlist	Difference	p value for gender* lottery winner/ *waitlist interaction term*
**Unadjusted Mean Score**								
For any reason	2.24	2.11	0.13	0.44	1.30	1.32	−0.02	0.91
Due to gender	0.49	0.58	−0.08	0.31	0.05	0.09	−0.04	0.22
Due to race	0.89	0.85	0.03	0.77	0.60	0.51	0.08	0.46
**Adjusted mean score**								
For any reason	2.16	2.07	0.10	0.62	1.35	1.38	−0.03	0.90
Due to gender	0.48	0.57	−0.10	0.28	0.04	0.09	−0.05	0.33
Due to race	0.83	0.83	0.00	0.98	0.62	0.57	0.05	0.70

Intent-to-treat analyses were used to estimate adjusted mean differences in Everyday Discrimination scores between lottery winners vs. waitlist. Hierarchical regression models account for students clustered within schools and included interaction terms between the main exposure (lottery winners vs. waitlist)

## Data Availability

Data and code for the analysis is available at this temporary link: at https://tinyurl.com/53r75r97. When the manuscript is published, we will update the link to a publicly accessible permanent link.

## Appendix A. Supplementary data

Supplementary data to this article can be found online at https://doi.org/10.1016/j.socscimed.2025.118923.
