# Supplementary material for: Striving and thriving: Gender differences in the effects of climbing the socioeconomic ladder on stress and discrimination
Source: Soc Sci Med. Author manuscript; Available in PMC 2026 Jul 1. (PMC13322100; doi:10.1016/j.socscimed.2025.118923)
Supplement: 1 [file NIHMS2189231-supplement-1.docx]

**Appendix**

**Table 6. Sensitivity analysis excluding race, language and birthplace as covariates: Differences (se) in stress between the lottery winners and waitlist groups among females and males.**

|  | **Females** | **Males** | **All** | ***p* value for gender*lottery winner/waitlist interaction term** |
| --- | --- | --- | --- | --- |
| **ASQ Stress at age 14-20** | 0.155** | 0.048 | 0.105** | 0.21 |
|  | (0.052) | (0.049) | (0.037) |  |
| Stress at home | 0.159** | -0.017 | 0.077* | 0.07 |
|  | (0.052) | (0.046) | (0.036) |  |
| Stress about school performance | 0.096* | 0.09 | 0.094** | 0.75 |
|  | (0.046) | (0.048) | (0.034) |  |
| Stress about romantic relationships | 0.018 | 0.021 | 0.02 | 0.53 |
|  | (0.043) | (0.046) | (0.033) |  |
| Stress of peer pressure | 0.149** | 0.02 | 0.089* | 0.14 |
|  | (0.054) | (0.047) | (0.037) |  |
| Stress of future uncertainty | 0.037 | 0.002 | 0.021 | 0.41 |
|  | (0.046) | (0.048) | (0.034) |  |
| Stress about the school/leisure conflict | 0.171** | 0.115* | 0.145** | 0.35 |
|  | (0.048) | (0.048) | (0.035) |  |
| Stress of financial pressure | 0.099* | 0.029 | 0.066 | 0.37 |
|  | (0.049) | (0.049) | (0.035) |  |
| **Perceived stress scale at age 21 and age 22** | 0.132* | -0.129 | 0.009 | 0.02 |
|  | (0.062) | (0.074) | (0.048) |  |

Table 3 Legend. Intent-to-treat analyses were used to estimate difference in stress between lottery winners vs. waitlist. Hierarchical regression models account for multiple observations over time and students clustered within schools and include interaction terms between the main exposure (lottery winners vs. waitlist) and gender. Standard error estimates were based on bootstrapping with 1000 repetitions. Significance levels *=5% **=1%.

**Table 7. Sensitivity analysis excluding race, language and birthplace as covariates: Everyday Discrimination Scores for the lottery winners and waitlist groups among females and males.**

|  | **Females** | | | | **Males** | | | |
| --- | --- | --- | --- | --- | --- | --- | --- | --- |
|  | **Lottery winner** | **Lottery Waitlist** | **Difference** | ***p* value for gender* lottery winner/waitlist interaction term** | **Lottery winner** | **Lottery Waitlist** | **Difference** | ***p value for gender* lottery winner/waitlist interaction term*** |
| **Unadjusted Mean Score** | | | | |  |  |  |  |
| For any reason | 2.24 | 2.11 | 0.13 | 0.44 | 1.30 | 1.32 | -0.02 | 0.91 |
| Due to gender | 0.49 | 0.58 | -0.08 | 0.31 | 0.05 | 0.09 | -0.04 | 0.22 |
| Due to race | 0.89 | 0.85 | 0.03 | 0.77 | 0.60 | 0.51 | 0.08 | 0.46 |
| **Adjusted mean score*** |  |  |  |  |  |  |  |  |
| For any reason | 2.16 | 2.06 | 0.10 | 0.62 | 1.35 | 1.38 | -0.02 | 0.90 |
| Due to gender | 0.48 | 0.57 | -0.09 | 0.29 | 0.05 | 0.10 | -0.05 | 0.35 |
| Due to race | 0.81 | 0.83 | -0.01 | 0.93 | 0.63 | 0.57 | 0.05 | 0.69 |

**Table 5 Legend.** Intent-to-treat analyses were used to estimate adjusted mean differences in Everyday Discrimination scores between lottery winners vs. waitlist. Hierarchical regression models account for students clustered within schools and included interaction terms between the main exposure (lottery winners vs. waitlist) and gender.
